# Psychosocial Burden, Multi-System Somatic Symptom Severity, and Weight-Related Stigma in Late Adolescents and Young Adults: A Cross-Sectional Survey from Romania

**DOI:** 10.3390/life16060969

**Published:** 2026-06-09

**Authors:** Raluca Maior, Hajnal Finta, Halit Tanju Besler, Elena Mardale, Simona Toncean, Vladimir Bacarea

**Affiliations:** 1 Department of Community Nutrition and Food Safety, George Emil Palade University of Medicine, Pharmacy, Science and Technology of Târgu Mureș, Gheorghe Marinescu Street No. 38, 540142 Târgu Mureș, Romania; raluca.maior@umfst.ro; 2 Department of Public Health and Health Management, George Emil Palade University of Medicine, Pharmacy, Science and Technology of Târgu Mureș, Gheorghe Marinescu Street No. 38, 540142 Târgu Mureș, Romania; 3 Department of Nutrition and Dietetics, Faculty of Health Sciences, Istanbul Aydın University, Beşyol Neighborhood, İnönü Street, No. 38, Küçükçekmece, 34295 Istanbul, Türkiye; halittanjubesler@aydin.edu.tr; 4 Doctoral School of Biomedical Sciences, University of Oradea, Universității Street No. 1, 410087 Oradea, Romania; elena.mardale@reginamaria.ro; 5 Department of Management, Dr. Eugen Nicoară Municipal Hospital, 545300 Reghin, Romania; s.boariu@yahoo.com; 6 Department of Research Methodology, George Emil Palade University of Medicine, Pharmacy, Science and Technology of Târgu Mureș, Gheorghe Marinescu Street No. 38, 540136 Târgu Mureș, Romania; vladimir.bacarea@umfst.ro

**Keywords:** psychosocial burden, weight stigma, body image, somatic symptom severity, perceived stress, BMI, late adolescents, cross-sectional survey, Romania

## Abstract

Evidence on the interplay between perceived stress, dietary behaviour, and weight-related psychosocial burden in Romanian young adults remains scarce. This cross-sectional study assessed associations between BMI, perceived stress, multi-system somatic symptom severity, and psychosocial burden in 117 participants aged 16 to 20 years (89.7% female; mean age 19.23 ± 0.74 years; mean BMI 22.66 ± 3.85 kg/m^2^), recruited by convenience sampling in Târgu Mureș, Romania, during June 2025. Non-parametric methods were used throughout. Female participants scored significantly higher than males across digestive (*p* < 0.001), neurological (*p* = 0.001), cutaneous (*p* = 0.014), and total symptom domains (*p* < 0.001), with a median total symptom score of 21.0 versus 3.0 in males. Perceived stress correlated positively with neurological (rS = 0.445), cardiovascular (rS = 0.350), digestive (rS = 0.316), and total symptom scores (rS = 0.401; all *p* < 0.001). BMI was not associated with somatic symptoms but correlated with weight-related stigma (rS = 0.391, *p* < 0.001). Emotional distress was prevalent regardless of weight status: 60.7% reported food-related guilt and 59.8% reduced self-confidence, yet only 6.0% had consulted a mental health professional. Stress management, nutritional counselling, and body image support should target young adults across all BMI categories.

## 1. Introduction

Adolescence and young adulthood constitute a developmental period during which dietary habits consolidate, exposure to psychological stress intensifies, and weight-related psychosocial concerns tend to reach peak prevalence [1,2]. The relationship between perceived stress and multi-system somatic symptom severity is well-documented across digestive, neurological, cardiovascular, and cutaneous domains, and is mediated through neuroendocrine and autonomic pathways [3]. Weight-related stigma is common in this age group and has been independently linked to emotional distress, social withdrawal, reduced self-confidence, and diminished engagement in health-promoting behaviours including physical activity [4,5,6,7]. Of particular clinical relevance is that the Psychological Burden associated with body weight is not confined to individuals classified as overweight or obese: food-related guilt and internalised weight stigma have been documented across the full BMI spectrum, including among normal-weight young adults [7].

Alongside stress and body weight concerns, dietary behaviour during adolescence has emerged as a determinant of both somatic health and psychological wellbeing. Diets characterised by high consumption of ultra-processed foods, refined carbohydrates, and fried products have been associated with increased somatic symptom burden, systemic low-grade inflammation, and a higher prevalence of depressive and anxiety symptomatology in young adult populations [8,9]. Conversely, adherence to a Mediterranean dietary pattern, characterised by regular intake of fruits, vegetables, legumes, whole grains, fish, fermented dairy products, and olive oil, has shown protective associations with both mental health outcomes and gastrointestinal function [10,11,12]. In Central and Eastern European adolescent populations, Mediterranean diet adherence is consistently low: KIDMED (Mediterranean Diet Quality Index for Children and Adolescents)-based data from Romanian children documented high adherence in only 17.8% of participants, compared with 68.1% among Romanian children residing in Italy [13,14]. Breakfast skipping, a behaviour with documented associations with depressive symptoms (pooled OR 1.39) and anxiety (OR 1.51) in meta-analytic data [15,16,17], is prevalent in this age group and frequently co-occurs with irregular meal timing and snacking patterns that further compromise dietary quality. Despite this evidence, population-level dietary pattern data from Romanian late adolescents remain scarce, and the relationship between dietary behaviour, perceived stress, somatic symptom severity, and weight-related psychosocial burden has not been examined in an integrated analytical framework within this specific regional sample.

A growing body of mechanistic research, primarily derived from animal models and selected clinical populations, has proposed the gut–brain axis as a theoretical framework linking psychological stress, neuroendocrine activation, and somatic symptom expression [2,16,17]. These findings provide a biological rationale for examining stress–symptom relationships at the population level; however, they rely on biomarker-level data including microbiome profiling, inflammatory markers, and intestinal permeability assessment, none of which were collected in the present study. The theoretical relevance of these pathways is therefore acknowledged as background context rather than as a testable hypothesis within this survey-based investigation.

Despite growing recognition of the interplay between perceived stress, body weight, and somatic and psychosocial health, integrative cross-sectional studies addressing these associations simultaneously in young Romanian adults remain scarce. The relative contribution of perceived stress versus BMI to symptom severity and psychological distress is particularly under-characterised in this population. The aim of this study is to quantify multi-system somatic symptom severity and psychosocial distress in a regional convenience sample of late adolescents and young adults in Târgu Mureș, Romania, to assess differences by sex and BMI category, and to compare the associations of perceived stress and BMI with these outcomes.

## 2. Materials and Methods

### 2.1. Study Design and Setting

This study used a cross-sectional survey design. Data collection took place in June 2025 in Târgu Mureș, central Romania, through a structured self-administered questionnaire. The study was conducted in accordance with the principles of the Declaration of Helsinki, and the research protocol received approval from the Ethics Committee of George Emil Palade University of Medicine, Pharmacy, Science and Technology of Târgu-Mureș, approval number 3746, approved 30 April 2025. Participants were recruited through convenience sampling from secondary school and university educational institutions. The questionnaire comprised 165 items completed independently by each participant.

Informed consent for participation was obtained from all subjects involved in the study. As data were collected through a fully anonymous, voluntary questionnaire with no identifying information recorded, written signatures were not required. All participants, including minors, received written information about the study’s objectives, voluntary nature, and guaranteed anonymity prior to participation. Voluntary completion of the questionnaire constituted consent to participate.

### 2.2. Participants

Inclusion criteria required participants to be between 16 and 20 years of age and to take part voluntarily. Individuals who submitted incomplete questionnaire responses or fell outside the specified age range were excluded from the analysis.

### 2.3. Measures

Anthropometric data included self-reported weight, height, and derived BMI, collected alongside lifestyle variables: physical activity level assessed on a 4-point ordinal scale (sedentary, low, moderate, very active), sleep duration and quality, and daily meal frequency. Perceived stress was rated on a 5-point Likert scale (1 = not at all stressed; 5 = extremely stressed).

The multi-system somatic symptom severity instrument was developed specifically for this study. No single validated questionnaire exists that simultaneously covers digestive, cutaneous, respiratory, neurological, and cardiovascular functional somatic complaints in a unified framework suitable for adolescent self-administration. Item content was therefore derived from three sources: (a) clinical symptom inventories used in functional somatic syndrome research, particularly the Patient Health Questionnaire-15 (PHQ-15) somatic symptom checklist and the Giessen Subjective Complaints List (GBB-24), which informed the selection of symptom domains and individual complaint items; (b) published epidemiological surveys of adolescent somatic symptom burden, which guided the inclusion of age-relevant complaints such as concentration difficulties, chronic fatigue, and postprandial fullness; and (c) clinical experience of the research team in nutritional consultation with young adult populations. The initial item pool of 42 candidate symptoms was reviewed for face validity, comprehensibility, and age-appropriateness by three academics (two in public health nutrition, one in research methodology) affiliated with the study institution. Five items were removed due to redundancy or ambiguity, yielding a final instrument of 37 items distributed across five clinically defined domains. Items were rated on a six-point severity scale (0 = no symptoms; 1 = very mild; 2 = mild; 3 = moderate; 4 = severe; 5 = very severe). This instrument has not been used in prior published research. Internal consistency was assessed using Cronbach’s alpha in the present sample (N = 117), with domain-level coefficients ranging from 0.632 (cardiovascular) to 0.882 (neurological), and overall instrument alpha of 0.895. No test–retest reliability or criterion validity data are currently available; these represent priorities for future validation work.

Multi-system somatic symptom severity was assessed across five domains using items rated on a 0–5 scale (0 = no symptoms; 5 = very severe symptoms), consistent with methodological approaches in published adolescent somatic symptom research [9,11,12]. All items evaluated symptoms experienced during the preceding four weeks.

Digestive domain (13 items, α = 0.798): bloating, gastric reflux and heartburn, nausea, vomiting, eructation, flatulence, constipation (fewer than three bowel movements per week or difficulty passing stool), diarrhoea (loose or watery stools more than three times daily), postprandial fullness, halitosis, urinary difficulties, and supplementary free-text items. Cutaneous domain (7 items, α = 0.736): eczema, spontaneous skin flushing or redness of the face, periorbital oedema and erythema, atopic dermatitis, pruritus, urticaria, and other skin symptoms. Respiratory domain (5 items, α = 0.797): chronic nasal congestion without infection, frequent sneezing, rhinorrhoea, breathing difficulties or shortness of breath, and chronic cough. Cardiovascular domain (5 items, α = 0.632): palpitations, orthostatic hypotension (dizziness or fainting upon standing), hypertensive episodes (sensation of pressure in head with rapid heart rate), musculoskeletal pain, and excessive perspiration. Neurological domain (7 items, α = 0.882): frequent headaches or migraines; dizziness or vertigo with balance disturbance; subjective cognitive difficulties described as “brain fog” (characterised by mental fatigue, reduced mental clarity, concentration difficulties, word-finding difficulty, and frequent forgetfulness, consistent with published characterisations of subjective brain fog in young adults); insomnia or nocturnal awakenings; irritability or abrupt mood changes; concentration problems; and chronic fatigue.

These neurological items capture functional somatic complaints within the neurocognitive spectrum and do not correspond to validated diagnostic criteria for neurological or psychiatric disorders. Brain fog is recognised as a subjective symptom reflecting how the brain responds to stress, sleep deprivation, and overall health rather than structural neurological damage.

Domain scores were computed as the sum of all item ratings. A total symptom score was calculated by summing all five domain scores.

The psychosocial burden battery was constructed de novo for this study, drawing on established conceptual frameworks in weight stigma research, body image theory and Social Impact assessment in obesity research. Item content was informed by three validated instruments that the battery does not replicate but from which thematic domains were adapted: the Weight Self-Stigma Questionnaire (WSSQ), which informed the stigma and internalisation items; the Impact of Weight on Quality of Life Questionnaire (IWQOL-Lite), which informed the Social Impact and avoidance domains; and the Sociocultural Attitudes Towards Appearance Questionnaire (SATAQ-), which informed the societal pressure and media items. The battery comprises 20 binary (yes/no) items distributed across seven conceptually distinct domains: (A) Psychological/Emotional Burden (5 items, range 0–5): shame related to body weight, guilt after food intake, reduced self-confidence due to physical appearance, social anxiety related to weight, and depressive feelings associated with body weight; (B) Social Impact (3 items, range 0–3): perceived effects of weight on personal relationships, academic or work performance, and participation in social activities; (C) Avoidance Behaviours (4 items, range 0–4): avoidance of beach or pool environments, gyms or sports facilities, photographs, and social events; (D) Stigma Experienced (2 items, range 0–2): self-reported discrimination and being the target of negative comments or jokes due to weight; (E) societal pressure and media (3 items, range 0–3); (F) societal beliefs (3 items, range 0–3); and (G) support and help-seeking resources (3 binary items). A Total Psychosocial Burden composite score was derived by summing domains A, B, and C only (range 0–12). Items were worded in Romanian and pilot-reviewed by three nutrition and public health academics for face validity and linguistic clarity prior to data collection. This battery has not been used in prior published research. Internal consistency in the present sample was acceptable to good: Emotional Burden α = 0.829, Social Impact α = 0.822, Avoidance α = 0.768, Stigma α = 0.764, Total Psychosocial Burden α = 0.889. Formal criterion validity assessment against the validated instruments listed above was not conducted and is recommended for future studies.

It is important to note that none of the psychological or behavioural constructs assessed in this study were measured using validated, standardised clinical instruments. Perceived stress was rated on a single-item five-point Likert scale rather than the ten-item Perceived Stress Scale (PSS-10) [18], which has demonstrated good reliability (Cronbach’s α = 0.78–0.85) and validity across large adolescent samples [19]. Depression and generalised anxiety were not assessed using the Patient Health Questionnaire-9 item version (PHQ-9) or the Generalised Anxiety Disorder-7 (GAD-7), which represent the current standard for brief depression and anxiety screening in non-clinical settings and have established cut-point scores for identifying clinically significant symptomatology in adolescent and young adult populations [20,21]. Eating disorder psychopathology was not evaluated using the Eating Attitudes Test-26 (EAT-26), the SCOFF questionnaire, or the Eating Disorder Inventory-3 (EDI-3), despite evidence that brief validated instruments such as SCOFF achieve sensitivity of 84.6% and specificity of 89.6% for detecting probable eating disorders in adolescent samples [22]. Body image disturbance was not assessed using validated instruments such as the Body Shape Questionnaire (BSQ), the Multidimensional Body-Self Relations Questionnaire (MBSRQ), or the Body Image Disturbance Questionnaire (BIDQ), all of which offer established normative data and psychometric validation in young adult populations [23,24]. The psychosocial burden battery used in this study was constructed de novo, drawing on established conceptual frameworks in weight stigma, body dissatisfaction, and Social Impact research, with demonstrated acceptable-to-good internal consistency (Cronbach’s α range 0.764–0.889). However, the absence of validated clinical instruments limits the ability to classify findings against published normative data, identify participants meeting diagnostic thresholds for clinical referral, compare results directly with the published literature using these instruments and assess measurement validity beyond internal consistency.

Internal consistency reliability was evaluated for all subscales using Cronbach’s alpha coefficient. Acceptable internal consistency was defined as alpha greater than 0.70, in accordance with established psychometric conventions [25,26]. The complete survey instrument is provided as Supplementary Material S1. The scoring algorithm for all composite scores, including domain-level symptom scales and psychosocial burden subscales, is detailed in Supplementary Material S2.

It should be noted that no composite dietary pattern score, summary dietary quality index, or formal dietary pattern classification was computed from the REAP (Rapid Eating Assessment for Patients) data. Unlike instruments such as the KIDMED index, which yield a single ordinal adherence score, or data-driven approaches such as principal component analysis or cluster analysis that derive empirical dietary patterns from food frequency data, the present study employed the REAP as a descriptive screening tool. Individual item frequencies were analysed and reported at the item level, with items classified post hoc into three interpretive categories (unhealthy dietary behaviours, healthy dietary behaviours and Mediterranean diet adherence indicators) based on established nutritional quality criteria. This descriptive approach was chosen for three reasons: (a) the four-point REAP response scale (often/always, sometimes, rarely/never, not applicable) does not lend itself to meaningful summation into a single composite index without arbitrary weighting assumptions; (b) item-level analysis preserves clinical interpretability, allowing identification of specific dietary behaviours (e.g., fried food consumption, breakfast skipping, olive oil use) associated with the somatic and psychosocial outcomes examined; and (c) the study population and sample size (N = 117) were not designed a priori for data-driven dietary pattern extraction (e.g., PCA or reduced-rank regression typically require larger samples and validated food frequency questionnaire data). The dietary analysis should therefore be understood as a descriptive dietary profile characterisation rather than a formal dietary pattern analysis in the epidemiological sense. This distinction is acknowledged as a limitation, and future studies with larger samples and validated food frequency questionnaires should consider computing composite dietary quality indices to enable dose–response analyses between dietary quality and health outcomes. Results are presented in Table 1.

### 2.4. Dietary Pattern Assessment

Dietary patterns were assessed using the Rapid Eating Assessment for Patients (REAP) [18], a validated brief dietary screening questionnaire comprising 43 items distributed across nine behavioural domains: dairy product intake; meat, fish, and protein sources; discretionary foods (sweets, salty snacks, fried foods, fast food); fruit and vegetable consumption; legumes, nuts, and seeds; fermented and probiotic foods; refined versus whole grain carbohydrate intake; beverage patterns (water, soft drinks, alcohol, coffee, tea, energy drinks); and meal frequency and timing regularity [27]. Each item was rated on a four-point frequency scale: often/always; sometimes; rarely/never; not applicable to me. Items were classified as reflecting healthy, unhealthy, or Mediterranean diet adherence patterns using established dietary quality criteria and the KIDMED framework [13]. Two single-item screening questions assessed self-reported appetite loss and excessive eating. All dietary data were obtained via self-administered questionnaire and are subject to social desirability and recall biases inherent to self-report methodology.

### 2.5. Statistical Analysis

All statistical analyses were conducted in Python 3.12 (SciPy 1.13, pandas 2.2) [11]. Normality of continuous variables was tested with the Shapiro–Wilk test. Descriptive statistics are presented as mean ± standard deviation (SD) and median with interquartile range (IQR) for continuous variables, and as frequency (n) with percentage (%) for categorical variables. Because most study variables did not follow normal distribution, non-parametric tests were applied throughout. Sex-based group differences were evaluated using the Mann–Whitney U test for non-normally distributed variables and the independent-samples t-test for height, which was the only normally distributed variable. Differences across BMI categories (underweight, normal weight, overweight, obese) were tested with the Kruskal–Wallis H test (df = 3); post hoc pairwise comparisons were performed using Mann–Whitney U with Bonferroni correction (k = 6 comparisons). Associations between continuous variables were quantified using the Spearman rank-order correlation coefficient (rS). Effect sizes for Mann–Whitney U tests were expressed as rank-biserial correlation. Chi-square tests (χ^2^, df = 3) were used to examine associations between categorical BMI classification and binary perception items. A two-sided p-value below 0.05 was set as the threshold for statistical significance. No missing data imputation was necessary, as complete responses were available for all analysed variables (N = 117).

Multiple linear regression analyses were conducted to examine independent associations between predictors and outcome variables after simultaneous adjustment for potential confounders. Predictors included in all regression models were sex, age (years), BMI (continuous, kg/m^2^), perceived stress (1–5 scale), physical activity level (1–4 ordinal), sleep duration (1–3 ordinal), education level (binary: secondary vs. university), and daily meal frequency (ordinal). Ordinary least squares estimation was applied. Model fit was evaluated using the F-statistic and coefficient of determination (R^2^). Unstandardised regression coefficients (B) with standard errors (SEs) and 95% confidence intervals are reported for each predictor.

To control for type I error inflation arising from multiple statistical comparisons, false discovery rate (FDR) correction was applied using the Benjamini–Hochberg procedure. This method controls the expected proportion of false positives among rejected null hypotheses and is appropriate for exploratory research involving correlated outcomes. FDR adjustment was applied separately to four families of tests: (1) Spearman correlations between BMI and 11 outcome variables; (2) Spearman correlations between perceived stress and the same 11 outcomes; (3) Kruskal–Wallis tests comparing BMI categories across 11 outcomes; and (4) chi-square tests of independence examining associations between BMI category and 17 binary psychosocial perception items. Adjusted p-values (pFDR) are reported alongside raw p-values in Results. Statistical significance after FDR correction was defined as pFDR < 0.05.

Sex-based group differences were evaluated using the Mann–Whitney U test. However, due to the substantial numerical imbalance between female (N = 105) and male (N = 12) participants, these specific comparative analyses should be treated as strictly exploratory and interpreted with caution, given the statistical fragility and limited statistical power inherent to the smaller subgroup.

Confidence intervals (95% CI) were calculated for key effect sizes (rank-biserial correlations) and primary Spearman rank-order correlation coefficients (p) using bootstrapping methods (1000 resamples) to provide interval estimation alongside exact *p*-values.

## 3. Results

### 3.1. Sample Characteristics

The sample comprised 117 participants (89.7% female) with a mean age of 19.23 years and a mean BMI of 22.66 kg/m^2^. Most were classified as normal weight (71.8%), with smaller proportions classified as overweight (12.8%), obese (7.7%), or underweight (7.7%). Detailed sample composition and lifestyle characteristics are presented in Table 2 and Table 3.

### 3.2. Distribution of Continuous Variables

Height was the only variable demonstrating a normal distribution (Shapiro–Wilk W = 0.981, *p* = 0.098). All remaining continuous variables, including BMI, all symptom domain scores, and perceived stress level, were non-normally distributed (all *p* < 0.001). The mean perceived stress level was 3.11 ± 0.95 (median 3.00). Neurological symptoms represented the highest symptom domain burden (mean 9.59 ± 7.69; median 8.00), followed by digestive symptoms (mean 7.45 ± 6.70; median 6.00). The total symptom score ranged from 0 to 100, with a mean of 24.19 ± 19.03. (Table 4).

### 3.3. Sex-Based Differences in Symptom Burden

Female participants demonstrated significantly higher symptom burden compared with male participants across digestive (U = 1034.0, *p* < 0.001), neurological (U = 996.0, *p* = 0.001), cutaneous (U = 887.5, *p* = 0.014), and total symptom domains (U = 1025.5, *p* < 0.001). These differences should be interpreted with caution given the considerable sex imbalance in the sample (N = 105 female, N = 12 male) and the resulting limited statistical power for male subgroup comparisons; all sex-based analyses are exploratory in nature. The median total symptom score was higher in females (21.0; IQR 12.0–35.0) than in males (3.0; IQR 0.25–10.25). Respiratory and cardiovascular symptom scores did not differ significantly by sex (*p* = 0.071 and *p* = 0.359, respectively). BMI was comparable between sexes (*p* = 0.156). Female participants also reported significantly higher Emotional Burden (*p* = 0.014), Avoidance Behaviours (*p* = 0.045), and Total Psychosocial Burden (*p* = 0.017) compared with male participants (Table 5).

### 3.4. BMI Category Differences in Psychosocial Burden

No somatic symptom domain differed significantly across BMI categories (all Kruskal–Wallis *p* > 0.05). Among psychosocial subscales, Stigma Experienced showed the strongest BMI category effect (H = 21.203, *p* < 0.001, ε^2^ = 0.162), with post hoc comparisons confirming that obese participants differed from normal-weight participants on both Stigma (p_adj < 0.001) and Social Impact (p_adj = 0.018). All participants classified as obese reported being targeted by negative weight-related comments, compared with 27.4% of normal-weight participants. Avoidance Behaviours showed a significant omnibus test (H = 8.456, *p* = 0.038), but no pairwise comparison survived Bonferroni correction. Full Kruskal–Wallis results and post hoc pairwise comparisons are presented in Table 6 and Table 7.

Post hoc comparisons with Bonferroni correction revealed that obese participants differed significantly from normal-weight participants on Stigma (p_adj < 0.001) and Social Impact (p_adj = 0.018) subscales. Notably, 100% of participants (N = 9/9) classified as obese reported being the target of negative comments or jokes about their weight, compared with 27.4% of normal-weight participants. BMI demonstrated statistically significant positive associations with four psychosocial outcomes after FDR correction: Emotional Burden (rS = +0.232, *p* = 0.012, pFDR = 0.033), Avoidance Behaviours (rS = +0.301, *p* = 0.001, pFDR = 0.005), Stigma Experienced (rS = +0.391, *p* < 0.001, pFDR < 0.001), and Total Psychosocial Burden (rS = +0.270, *p* = 0.003, pFDR = 0.012). Correlations with symptom domain scores were not statistically significant after FDR correction.

Perceived stress showed significant positive correlations with 10 of 11 outcome variables after FDR correction, with the strongest associations for neurological symptoms (rS = 0.445, pFDR < 0.001) and total symptom score (rS = 0.401, pFDR < 0.001); only cutaneous symptoms were not significantly associated (pFDR = 0.414). In contrast, BMI correlated significantly with psychosocial outcomes only: stigma (rS = 0.391, pFDR < 0.001), avoidance (rS = 0.301, pFDR = 0.005), and Total Psychosocial Burden (rS = 0.270, pFDR = 0.012), but not with any somatic symptom domain (all pFDR > 0.05). Physical activity level was not associated with any outcome (all *p* > 0.05). Complete correlation coefficients with raw and FDR-adjusted p-values are presented in Table 8.

### 3.5. Psychosocial Burden Profiles and Help-Seeking Behaviour

Emotional Burden items were prevalent across all BMI categories. Guilt after eating was endorsed by 60.7%, reduced self-confidence by 59.8%, and shame by 53.0% of participants. Social anxiety related to weight was reported by 38.5% and depressive feelings by 27.4%. Among Avoidance Behaviours, avoidance of photographs was most common (29.9%), followed by beach or pool avoidance (27.4%) and gym avoidance (17.1%). Despite this burden, only 6.0% had consulted a mental health professional about weight-related concerns, while 61.5% reported receiving support from family or friends. Detailed frequencies for all binary perception items are available in Supplementary Material S1.

Avoidance of photography was the most common weight-related Avoidance Behaviour (29.9%), followed by avoidance of beach and pool environments (27.4%) and gym facilities (17.1%). Despite the high prevalence of psychosocial burden, only 6.0% of participants had consulted a mental health professional regarding weight-related concerns. In contrast, 61.5% reported receiving support from family or friends, and 14.5% had participated in a structured weight management programme.

Societal pressure awareness was high: 72.6% of participants agreed that media promotes unrealistic body image standards, and 64.1% felt pressure to conform to societal appearance norms. Among societal beliefs, 45.3% perceived overweight individuals as lazier and 44.4% as less healthy than the general population.

### 3.6. Multivariable Regression Analyses

To examine independent contributions of multiple covariates to symptom and psychosocial outcomes simultaneously, four multivariable linear regression models were estimated. Predictors entered in all models were sex, age, BMI, perceived stress, physical activity level, sleep duration, education level, and daily meal frequency. Results are presented in Table 8.

For total symptom score (Model 1), the overall model was statistically significant (F(8, 108) = 3.015, *p* = 0.004, R^2^ = 0.183). After adjustment for all covariates, female sex (B = +14.37, 95% CI [+2.15, +26.58], *p* = 0.022) and perceived stress (B = +6.62, 95% CI [+2.82, +10.42], *p* = 0.001) remained independently and significantly associated with higher total symptom burden. BMI did not contribute significantly to the model after covariate adjustment (*p* = 0.822).

For Total Psychosocial Burden (Model 2; F(8, 108) = 3.723, *p* < 0.001, R^2^ = 0.216), four predictors were independently significant: female sex (B = +2.64, *p* = 0.020), BMI (B = +0.23, *p* = 0.006), perceived stress (B = +0.76, *p* = 0.031), and university-level education, which showed an inverse association (B = −1.43, *p* = 0.033). This inverse association may reflect greater access to body-positive educational environments or higher health literacy, though this interpretation remains speculative without further data.

Model 3 for Stigma Experienced (F(8, 108) = 5.451, *p* < 0.001, R^2^ = 0.288) identified BMI as the sole independent significant correlate (B = +0.110, 95% CI [+0.073, +0.147], *p* < 0.001), with no other predictor reaching significance after adjustment. This finding indicates that the association between BMI and experienced weight stigma is robust to confounding by sex, age, stress, activity level, sleep, education, and meal frequency.

For neurological symptom score (Model 4; F(8, 108) = 4.556, *p* < 0.001, R^2^ = 0.252), perceived stress was the only significant independent correlate (B = +3.09, 95% CI [+1.62, +4.56], *p* < 0.001). Female sex showed a marginal non-significant trend (B = +4.21, *p* = 0.080). BMI was not associated with neurological symptom scores after adjustment (Table 9).

### 3.7. Dietary Pattern Analysis

Analysis of REAP questionnaire responses from all 117 participants revealed a mixed dietary profile combining prevalent unhealthy dietary behaviours, moderate intake of protective food groups, and markedly low Mediterranean diet adherence. Complete frequency data for all assessed items are presented in Table 10.

Unhealthy dietary patterns were highly prevalent. Several healthy dietary behaviours were also identified. Mediterranean diet adherence was markedly low across all three key indicators examined. The convergence of low olive oil, low fermented dairy, low fish, and low whole grain consumption confirms poor overall Mediterranean diet adherence in this central Romanian adolescent cohort, consistent with patterns documented among non-Mediterranean Eastern European university students [28,29]. Beverage analysis revealed that coffee was the most consumed drink (70.1% at least sometimes), followed by sweetened beverages (58.1%) and alcohol (34.2% at least sometimes)—Table 10.

## 4. Discussion

This cross-sectional survey captured a single temporal snapshot and cannot establish causal direction or mechanistic pathways. All associations reported below may reflect bidirectional relationships, shared third-variable influences such as neuroticism or health anxiety, or reporting biases inherent to self-administered questionnaires. With that constraint in mind, three findings stand out.

First, perceived stress was the variable most consistently associated with somatic symptom severity across domains: neurological (rS = 0.445), cardiovascular (rS = 0.350), digestive (rS = 0.316), and total symptom score (rS = 0.401) [8,9,12]. The neurological domain showed the strongest correlation, which mirrors survey-based evidence linking perceived stress to headache, concentration difficulties, and sleep disruption in comparable adolescent samples [14,15]. Neuroendocrine pathways connecting sustained stress to gastrointestinal and autonomic dysregulation have been proposed in clinical research [12,13], but these frameworks provide theoretical context only; testing them would require biological measurements unavailable in this study.

Second, BMI did not correlate with any somatic symptom domain but was the sole independent factor significantly associated with Stigma Experienced (rS = 0.391), Avoidance Behaviours (rS = 0.301), and psychosocial burden (rS = 0.270). The absence of a BMI–symptom link is less surprising when considering that 71.8% of participants were normal-weight and that metabolic consequences of excess adiposity typically take years to manifest clinically [16,17,18]. The sex differences in symptom reporting, with female participants scoring substantially higher across digestive, neurological, cutaneous, and total domains, are consistent with epidemiological data on sex-specific somatic symptom patterns and stress reactivity [10,19,20]. These sex-stratified analyses remain exploratory given the severe imbalance in the sample (105 females vs. 12 males).

Third, emotional distress related to food and body image was widespread regardless of weight status. Guilt after eating was endorsed by 60.7% of participants, reduced self-confidence by 59.8%, and shame by 53.0%, including among normal-weight individuals. This pattern fits the concept of internalised weight stigma operating independently of actual body weight [3,21]. Yet only 6.0% had consulted a psychologist about weight-related concerns, pointing to a gap between Psychological Burden and mental health service use that has been documented repeatedly in young adult populations [30]. It is worth noting that these Emotional Burden items were not derived from validated clinical instruments. Standardised tools such as the BSQ [31] or MBSRQ [32] would have allowed comparison against normative cut-points, and validated eating disorder screens (EAT-26, SCOFF) [33,34] would clarify whether the appetite disturbances observed (10.3% appetite loss, 8.5% excessive eating) reflect subclinical pathology or clinically significant symptomatology. Physical activity level was not associated with psychosocial burden in this sample, probably reflecting the narrow range of activity levels in a predominantly student cohort; wider variability has yielded protective associations in other studies [23].

### 4.1. Methodological Constraints

Common-method variance is a concern when the same respondent rates both predictor and outcome variables using similar response formats. Shared measurement error, negative affectivity and acquiescence bias can inflate correlation coefficients beyond what true associations would produce. The study also lacked biological measurements: no inflammatory markers (CRP, IL-6, TNF-α), intestinal permeability indicators (zonulin, LBP), or neuroendocrine measures (salivary cortisol) were collected. Without these data, it is not possible to determine whether the stress–somatic associations documented here operate through inflammatory or gut–brain pathways, or whether they primarily reflect shared variance in self-reported distress. The dietary data, particularly the high fried food prevalence and low Mediterranean adherence, add biological plausibility to an inflammatory mediation model, but plausibility does not constitute empirical confirmation. The present findings are best understood as hypothesis-generating observations that require prospective designs with concurrent biological sampling to move toward mechanistic evidence.

### 4.2. Dietary Patterns

The REAP questionnaire revealed a Western dietary profile: 77.8% consumed refined carbohydrates at least sometimes, 76.9% fried foods, and 62.4% skipped breakfast at least sometimes. Mediterranean diet adherence was low by all three indicator criteria (extra virgin olive oil regular use: 26.5%; fermented dairy ≥ 3 times/week: 36.8%; fresh fish ≥ 1–2 times/week: 16.2%), consistent with KIDMED data from Romanian children (17.8% high adherence in Romania vs. 68.1% among Italian-resident Romanian peers) [26,27,28] and with patterns among non-Mediterranean Eastern European students [13].

These dietary findings are relevant to the somatic and psychosocial profiles documented. A UK Biobank cohort study (N = 140,728) linked frequent fried food consumption to 12% higher anxiety and 7% higher odds of depression through acrolein-mediated neuroinflammation [29], and a meta-analysis of 17 prospective studies (N = 562,445) associated the highest fried food category with 28% higher cardiovascular event risk [30]. Breakfast omission has been linked to depressive symptoms (pooled OR 1.39, 95% CI 1.34–1.44) and anxiety (OR 1.51, 95% CI 1.25–1.77) in a meta-analysis of approximately 399,550 participants [31,32,33,34,35], with a recent scoping review confirming these associations specifically in adolescents [36]. Umbrella reviews have provided class-I evidence linking ultra-processed food patterns to adverse mental health outcomes (common mental disorder OR 1.53; depressive symptoms OR 1.44) and Mediterranean adherence to protective associations [24,37,38]. Whether improving dietary patterns would reduce symptom burden in this population cannot be answered with cross-sectional data and would require randomised controlled trials.

Low fermented dairy consumption (36.8% at the recommended frequency) is relevant to gut–brain axis frameworks, since regular intake at this level has been associated with enrichment of Lactobacillus and Bifidobacterium genera and improved barrier function markers [39,40]. Eating behaviour disturbances were present in roughly one-fifth of participants, consistent with European data reporting 22% probable eating disorder prevalence using the SCOFF questionnaire [41]; the co-occurrence of appetite dysregulation and Western dietary patterns is consistent with evidence linking weight stigma to disordered eating behaviours [42], though directionality cannot be established. Coffee (70.1%), alcohol (34.2%), and energy drinks (25.6%) were consumed at least sometimes, the alcohol figure aligning with ESPAD surveillance data for Romanian adolescents [43]. Energy drink consumption is specifically relevant given documented associations with sleep disruption, anxiety, and palpitations in young people [44], symptoms well-represented in the neurological and cardiovascular domains of this study.

### 4.3. Limitations

The findings of the present study must be interpreted in light of several critical methodological constraints. First, the cross-sectional design does not permit causal inference; all reported associations are strictly correlational and may reflect bidirectional relationships or shared third-variable influences. Furthermore, data were entirely self-reported, introducing potential recall and social desirability biases.

Crucially, anthropometric parameters were not objectively measured. Validation studies in adolescent and young adult samples demonstrate that self-report typically underestimates BMI by an average of 0.23–0.70 kg/m^2^, with a significantly stronger underreporting bias observed among young women, individuals in higher-BMI categories, and those experiencing elevated body dissatisfaction [45]. Because our analysis examines BMI, body image distress, and weight-related stigma simultaneously, this systematic underestimation introduces a potential risk of circularity and conservative bias, which may artificially constrain the true variance and weaken the observable statistical relationships between objective body mass and psychological outcomes.

Another consequential methodological constraint is the complete absence of biological specimen collection. No blood, saliva, stool, or urine samples were obtained, and no objective physiological measurements were performed. Consequently, the inflammatory and gut–brain axis pathways discussed as theoretical context cannot be empirically examined with the available data. Specifically, five categories of biomarkers would have enhanced the biological interpretability of these findings: (a) high-sensitivity C-reactive protein (hsCRP) as a marker of systemic low-grade inflammation independently linked to perceived stress and somatic symptom severity in youth [46,47]; (b) IL-6 and TNF-α as pro-inflammatory cytokines with established roles in sickness behaviour and central sensitisation, directly relevant to the neurological and cardiovascular symptom patterns observed [48,49]; (c) zonulin as a serum marker of intestinal barrier integrity that would have enabled assessment of whether the high fried food consumption and low Mediterranean adherence documented here relate to intestinal hyperpermeability [50]; (d) lipopolysaccharide-binding protein (LBP) reflecting diet-induced endotoxaemia associated with ultra-processed food intake [51,52]; and (e) salivary or hair cortisol providing objective verification of hypothalamic–pituitary–adrenal (HPA) axis reactivity beyond self-reported stress ratings [53]. The conclusions of this study therefore remain at the epidemiological level: the observed associations are statistically robust but generate rather than test mechanistic hypotheses.

Furthermore, several measurement limitations affect the specificity and comparability of our findings. The psychological and behavioural constructs assessed in this study were not measured using extensive, validated clinical batteries. While multi-item psychometric tools capture greater clinical nuance, the extensive public health literature confirms that single-item global stress measures possess satisfactory convergent validity and strong predictive capacity for functional somatic complaints, while mitigating questionnaire length and survey fatigue in educational settings. Nevertheless, the single-item design precluded the assessment of cognitive appraisal or specific coping resources. Similarly, depression and generalised anxiety were not screened with standardised tools like the PHQ-9 [54] or GAD-7 [55], which would have enabled statistical control for psychiatric comorbidity and direct comparison with published European adolescent data [56,57]. Eating disorder psychopathology was captured by two brief screening items rather than the EAT-26 [42] or SCOFF [56], and body image disturbance was not assessed using the BSQ [53], MBSRQ [58], or BIDQ [59], precluding distinction between subclinical weight-related worry and clinically significant body image pathology [46].

The neurological symptom domain, which assessed seven functional complaints (headache, dizziness, brain fog, insomnia, mood instability, concentration problems, fatigue) using severity ratings, lacked phenomenological characterisation. Crucially, no formal exclusion criterion for pre-existing neurological or psychiatric diagnoses was applied, meaning that elevated neurological symptom scores may reflect either stress-related functional symptoms or pre-existing clinical conditions, limiting the specificity of this domain. This lack of systematic assessment extends to seven categories of confounding variables: (1) psychiatric comorbidity; (2) clinical eating disorders; (3) medication use, including psychotropics and oral contraceptives; (4) substance use, including smoking and cannabis; (5) chronic gastrointestinal conditions such as IBS, for which symptoms but not formal diagnoses were recorded; (6) detailed socioeconomic indicators beyond the brief self-rating employed; and (7) hormonal factors including menstrual cycle phase and thyroid function. Residual confounding from these unmeasured variables limits the interpretability of all reported associations.

Finally, the generalizability of the present findings is constrained by the recruitment strategy and sample characteristics. Data collection was restricted to a single institutional and urban context in Târgu Mureș, Romania, utilising a convenience sample of secondary school and university students. Consequently, this cohort may not adequately represent the diverse socioeconomic, cultural, or geographic backgrounds of the broader Romanian youth population, particularly those residing in rural areas or those outside the formal educational system. Furthermore, the sample exhibits a highly imbalanced sex distribution, with female participants constituting 89.7% of the cohort (N = 105 females vs. N = 12 males). This imbalance is an inherent artefact of the convenience sampling approach, reflecting a well-documented higher propensity among young women to voluntarily participate in surveys addressing body weight and somatic health.

Consequently, while statistical power was adequate for the primary multivariable regression models (power > 0.96 for all models), it was insufficient for secondary sex-based comparisons. The sex imbalance yielded an achieved power of approximately 0.72 for the total symptom score sex comparison—falling below the conventional 0.80 threshold—with lower power for individual domain comparisons, while the obese male subgroup (N = 1) is entirely non-informative. Therefore, all sex-stratified findings must be treated as strictly exploratory, and the male subgroup results cannot be generalised—Table 11.

Nevertheless, this skewed distribution does not fully invalidate the study’s core findings regarding weight-related psychosocial burden and stress. Young females represent the precise demographic subgroup at the highest epidemiological risk for internalised weight stigma, body shape dissatisfaction, and functional somatic syndromes. Thus, while the findings cannot be generalised to young males or the national population, they provide highly valid, concentrated insights into the specific regional subsegment that is clinically most vulnerable to the synergistic effects of perceived stress and body image distress.

Future research should employ stratified random sampling designs across multiple regions to confirm these trends on a national scale, utilise balanced sex representation with a priori power calculations, and administer a standardised validated battery alongside objective anthropometric measurements and concurrent biological sampling (including hsCRP, IL-6, zonulin, and salivary cortisol) to enable formal clinical classification, control for psychiatric comorbidity, and empirical testing of the mechanistic hypotheses generated by the present findings.

## 5. Conclusions

In this regional cohort of late adolescents and young adults, perceived stress emerged as a prominent correlate of multi-system somatic symptom burden, showing consistent associations with neurological, cardiovascular, digestive, and total symptom domains. Objective BMI was not associated with somatic symptom severity but correlated significantly with weight-related stigma, situational avoidance, and Social Impact. Weight-related psychosocial burden and body image concerns were highly prevalent across the cohort regardless of weight status, highlighting a critical gap in accessible mental health support within nutritional contexts.

These exploratory findings suggest the utility of integrating stress management, body image counselling, and anti-stigma approaches into conventional nutritional and weight management practice. Given the cross-sectional nature of this survey, these associations remain at the hypothesis-generating level. Future prospective studies incorporating concurrent biological sampling—specifically hsCRP, IL-6, zonulin, and salivary cortisol—alongside standardised clinical instruments are required to establish temporal relationships and confirm the potential pathways linking perceived stress to multi-system somatic burden.

## Figures and Tables

**Table 1:** Internal consistency reliability (Cronbach’s alpha) for symptom domain and psychosocial subscales (N = 117).

Scale	No. of Items	Response Format	Cronbach’s Alpha	Interpretation
Psychosocial Subscales				
Emotional Burden	5	Binary (Yes/No)	0.829	Good
Social Impact	3	Binary (Yes/No)	0.822	Good
Avoidance Behaviours	4	Binary (Yes/No)	0.768	Acceptable
Stigma Experienced	2	Binary (Yes/No)	0.764	Acceptable
Total Psychosocial Burden (A + B + C)	12	Binary (Yes/No)	0.889	Excellent
Somatic Symptom Domain Scales				
Digestive	13	Likert 0–5	0.798	Acceptable
Cutaneous	7	Likert 0–5	0.736	Acceptable
Respiratory	5	Likert 0–5	0.797	Acceptable
Neurological	7	Likert 0–5	0.882	Good
Cardiovascular	5	Likert 0–5	0.632	Questionable

Note. Cronbach’s alpha interpretation: excellent ≥ 0.90; good = 0.80–0.89; acceptable = 0.70–0.79; questionable = 0.60–0.69; poor < 0.60 (Binary items coded da = 1, nu = 0. Psychosocial subscales computed as sum of binary items within each domain. Somatic symptom scales computed as sum of Likert-rated items (0–5) within each domain. Total Psychosocial Burden = sum of domains A (Emotional Burden) + B (Social Impact) + C (Avoidance Behaviours); range 0–12. All alpha values computed from complete dataset (N = 117).

**Table 2:** Sample composition (N = 117).

Variable	Category	N	%
Sex	Female	105	89.7
	Male	12	10.3
BMI Category	Underweight (<18.5 kg/m^2^)	9	7.7
	Normal weight (18.5–24.9 kg/m^2^)	84	71.8
	Overweight (25.0–29.9 kg/m^2^)	15	12.8
	Obese (≥30.0 kg/m^2^)	9	7.7
Education Level	Secondary school	39	33.3
	University	77	65.8

BMI = body mass index.

**Table 3:** Lifestyle characteristics (N = 117).

Variable	Category	N	%
Physical Activity	Sedentary	13	11.1
	Low (1–3 days/week)	51	43.6
	Moderately active (3–5 days/week)	44	37.6
	Very active (6–7 days/week)	9	7.7
Sleep Duration	<6 h/night	24	20.5
	6–8 h/night	82	70.1
	8–10 h/night	11	9.4
Daily Meals	1–2 meals/day	30	25.6
	3 meals/day	64	54.7
	4–5 meals/day	18	15.4

**Table 4:** Descriptive statistics for continuous variables (N = 117).

Variable	N	Mean ± SD	Median	IQR	Min	Max
Age (years)	117	19.23 ± 0.74	19.00	19.00–20.00	16	20
BMI (kg/m^2^)	117	22.66 ± 3.85	22.10	19.92–24.38	14.53	35.43
Perceived Stress (1–5)	117	3.11 ± 0.95	3.00	3.00–4.00	1	5
Digestive Score	117	7.45 ± 6.70	6.00	3.00–10.00	0	36
Neurological Score	117	9.59 ± 7.69	8.00	4.00–14.00	0	31
Cutaneous Score	117	2.64 ± 4.24	1.00	0.00–3.00	0	19
Cardiovascular Score	117	2.34 ± 2.99	1.00	0.00–3.00	0	17
Total Symptom Score	117	24.19 ± 19.03	20.00	12.00–34.00	0	100

SD = standard deviation; IQR = interquartile range; BMI = body mass index.

**Table 5:** Sex-based comparison of symptom and psychosocial burden scores (N = 117).

Variable	Female Mean ± SD (N = 105)	Male Mean ± SD (N = 12)	*p*-Value	Test	Sig.
Digestive Score	8.06 ± 6.77	2.17 ± 2.52	<0.001	MWU	***
Neurological Score	10.28 ± 7.66	3.58 ± 5.07	0.001	MWU	**
Cutaneous Score	2.88 ± 4.39	0.58 ± 1.51	0.014	MWU	*
Total Symptom Score	25.90 ± 18.89	9.25 ± 13.23	<0.001	MWU	***
Emotional Burden	2.53 ± 1.83	1.17 ± 1.75	0.014	MWU	*
Avoidance Behaviours	0.95 ± 1.30	0.25 ± 0.62	0.045	MWU	*
Total Psychosocial Burden	4.06 ± 3.55	1.75 ± 2.56	0.017	MWU	*

MWU = Mann–Whitney U test. *** *p* < 0.001; ** *p* < 0.01; * *p* < 0.05.

**Table 6:** Kruskal–Wallis H Test: BMI category vs symptom and psychosocial burden scores (N = 117).

Outcome Variable	Underw. Md	Normal Md	Overw. Md	Obese Md	H	*p*	pFDR	Sig.
Somatic Symptom Domains								
Digestive Score	7.00	5.00	9.00	5.00	4.010	0.260	0.429	ns
Cutaneous Score	1.00	0.50	2.00	0.00	2.765	0.429	0.686	ns
Respiratory Score	2.00	1.00	1.00	3.00	4.056	0.256	0.429	ns
Neurological Score	6.00	8.00	12.00	5.00	3.052	0.384	0.560	ns
Cardiovascular Score	1.00	1.00	2.00	1.00	1.033	0.793	0.793	ns
Total Symptom Score	22.0	19.5	20.0	19.0	1.362	0.714	0.793	ns
Psychosocial Burden Subscales								
Emotional Burden	2.0	2.0	3.0	4.0	4.509	0.211	0.388	ns
Social Impact	0.0	0.0	0.0	1.0	9.498	0.023	0.085	ns †
Avoidance Behaviours	0.0	0.0	1.0	1.0	8.456	0.038	0.105	ns †
Stigma Experienced	0.0	0.0	0.0	2.0	21.203	<0.001	<0.001	***
Total Psychosocial Burden	3.0	3.0	4.0	7.0	7.350	0.062	0.171	†

Md = median. n values per group: underweight = 9, normal weight = 84, overweight = 15, obese = 9. H = Kruskal–Wallis statistic (df = 3). p FDR = Benjamini–Hochberg false discovery rate-corrected *p*-value (Family 3: 11 tests). *ε*^2^ (epsilon-squared) effect size reported for significant omnibus tests: *Stigma ε*^2^ = 0.162 (large). *** *p* < 0.001; † marginal trend (*p* < 0.10 but p FDR > 0.05); ns = not significant. BMI categories: underweight < 18.5; normal 18.5–24.9; overweight 25.0–29.9; obese ≥ 30.0 kg/m^2^.

**Table 7:** Post hoc pairwise comparisons (Mann–Whitney U, Bonferroni correction): significant Kruskal–Wallis subscales.

Subscale	Pairwise Comparison	U	p_aej_ (Bonf., k = 6)	Sig.
Stigma Experienced (H = 21.203, *p* < 0.001, ε^2^ = 0.162)				
	Normal weight vs. Obese	76	<0.001	***
	Underweight vs. Obese	8	0.012	*
	Overweight vs. Obese	38	0.312	ns
	Normal weight vs. Overweight	482	0.528	ns
	Normal weight vs. Underweight	306	1.000	ns
	Underweight vs. Overweight	52	1.000	ns
Social Impact (H = 9.498, *p* = 0.023, ε^2^ = 0.064)				
	Normal weight vs. Obese	200	0.018	*
	Underweight vs. Obese	20	0.307	ns
	Overweight vs. Obese	48	1.000	ns
	Normal weight vs. Overweight	538	1.000	ns
	Normal weight vs. Underweight	355	1.000	ns
	Underweight vs. Overweight	58	1.000	ns
Avoidance Behaviours (H = 8.456, *p* = 0.038)—no pairs significant after correction				
	All pairwise comparisons	—	≥0.197 (all ns after Bonf.)	ns

Bonferroni correction applied for k = 6 pairwise comparisons per subscale. *** *p _aej_* < 0.001; * *p _aej_* < 0.05; ns = not significant after correction. Avoidance Behaviours: omnibus test significant (H = 8.456, *p* = 0.038), but no pairwise comparison reached significance after Bonferroni correction; threshold effect is present but cannot be localised to a specific group contrast. Effect size for Stigma: *ε*^2^ = 0.162, large. Effect size for Social Impact: *ε*^2^ = 0.064, small-to-medium.

**Table 8:** Spearman rank-order correlations: BMI and perceived stress vs outcome variables (N = 117).

Outcome Variable	BMI rS	BMI *p*	BMI pFDR	Stress rS	Stress *p*	Stress pFDR
Somatic Symptom Domains						
Digestive Score	−0.044	0.640	0.793	+0.316	<0.001	0.002 **
Cutaneous Score	−0.020	0.828	0.828	+0.076	0.414	0.414
Respiratory Score	−0.021	0.823	0.828	+0.193	0.037	0.102
Neurological Score	+0.114	0.221	0.388	+0.445	<0.001	<0.001 ***
Cardiovascular Score	−0.001	0.995	0.995	+0.350	<0.001	<0.001 ***
Total Symptom Score	+0.053	0.574	0.793	+0.400	<0.001	<0.001 ***
Psychosocial Burden Subscales						
Emotional Burden	+0.232	0.012	0.033 *	+0.290	0.002	0.011 **
Social Impact	+0.194	0.036	0.099	+0.243	0.008	0.029 *
Avoidance Behaviours	+0.301	0.001	0.005 **	+0.235	0.011	0.030 *
Stigma Experienced	+0.391	<0.001	<0.001 ***	+0.189	0.041	0.113
Total Psychosocial Burden	+0.270	0.003	0.012 **	+0.295	0.001	0.011 **

rS = Spearman rank correlation coefficient. *p* = two-tailed significance (raw). pFDR = Benjamini–Hochberg false discovery rate-corrected *p*-value; BMI correlations corrected within Family 1 (11 tests); perceived stress correlations within Family 2 (11 tests). Significance after FDR correction: *** p FDR < 0.001; ** p FDR < 0.01; * p FDR < 0.05. Effect sizes small = 0.10–0.29; moderate = 0.30–0.49; large ≥ 0.50. Physical activity level was not significantly correlated with any psychosocial subscale or BMI (all *p* > 0.05, all p FDR > 0.05).

**Table 9:** Multivariable linear regression: independent correlates of symptom and psychosocial burden scores (N = 117).

Predictor	B	SE	t	p	95% CI	Sig.
Model 1: Total Symptom Score R^2^ = 0.183; Adj. R^2^ = 0.122; F(8, 108) = 3.015, *p* = 0.004						
Sex (female = 1)	+14.365	6.164	2.330	0.022	[+2.15, +26.58]	*
Age (years)	+0.432	2.386	0.181	0.857	[−4.30, +5.16]	ns
BMI (kg/m^2^)	+0.103	0.458	0.225	0.822	[−0.81, +1.01]	ns
Perceived Stress	+6.621	1.916	3.456	0.001	[+2.82, +10.42]	***
Physical Activity	+0.184	2.276	0.081	0.936	[−4.33, +4.70]	ns
Sleep Duration	+1.290	3.628	0.355	0.723	[−5.90, +8.48]	ns
Education (university = 1)	−3.478	3.652	−0.952	0.343	[−10.72, +3.76]	ns
Daily Meals	−0.259	2.674	−0.097	0.923	[−5.56, +5.04]	ns
Model 2: Total Psychosocial Burden R^2^ = 0.216; Adj. R^2^ = 0.158; F(8, 108) = 3.723, *p* < 0.001						
Sex (female = 1)	+2.642	1.118	2.364	0.020	[+0.43, +4.86]	*
Age (years)	−0.246	0.433	−0.569	0.571	[−1.10, +0.61]	ns
BMI (kg/m^2^)	+0.233	0.083	2.798	0.006	[+0.07, +0.40]	**
Perceived Stress	+0.758	0.347	2.181	0.031	[+0.07, +1.45]	*
Physical Activity	+0.102	0.413	0.247	0.806	[−0.72, +0.92]	ns
Sleep Duration	+0.029	0.658	0.044	0.965	[−1.28, +1.33]	ns
Education (university = 1)	−1.428	0.662	−2.156	0.033	[−2.74, −0.12]	*
Daily Meals	−0.252	0.485	−0.520	0.604	[−1.21, +0.71]	ns
Model 3: Stigma Experienced R^2^ = 0.288; Adj. R^2^ = 0.235; F(8, 108) = 5.451, *p* < 0.001						
Sex (female = 1)	+0.384	0.251	1.533	0.128	[−0.11, +0.88]	ns
Age (years)	−0.034	0.097	−0.352	0.726	[−0.23, +0.16]	ns
BMI (kg/m^2^)	+0.110	0.019	5.922	< 0.001	[+0.07, +0.15]	***
Perceived Stress	+0.080	0.078	1.024	0.308	[−0.08, +0.23]	ns
Physical Activity	−0.031	0.093	−0.337	0.737	[−0.22, +0.15]	ns
Sleep Duration	+0.171	0.148	1.158	0.249	[−0.12, +0.46]	ns
Education (university = 1)	−0.234	0.148	−1.573	0.119	[−0.53, +0.06]	ns
Daily Meals	+0.007	0.109	0.065	0.948	[−0.21, +0.22]	ns
Model 4: Neurological Symptom Score R^2^ = 0.252; Adj. R^2^ = 0.197; F(8, 108) = 4.556, *p* < 0.001						
Sex (female = 1)	+4.206	2.382	1.766	0.080	[−0.52, +8.93]	†
BMI (kg/m^2^)	+0.094	0.177	0.530	0.597	[−0.26, +0.45]	ns
Perceived Stress	+3.090	0.740	4.173	< 0.001	[+1.62, +4.56]	***
Physical Activity	−0.744	0.879	−0.846	0.399	[−2.49, +1.00]	ns
Sleep Duration	+0.118	1.402	0.084	0.933	[−2.66, +2.90]	ns
Education (university = 1)	−0.811	1.411	−0.575	0.567	[−3.61, +1.99]	ns
Daily Meals	−1.003	1.033	−0.971	0.334	[−3.05, +1.05]	ns

Note. B = unstandardised regression coefficient; SE = standard error; 95% CI = confidence interval for B. *** *p* < 0.001; ** *p* < 0.01; * *p* < 0.05; † marginal trend (*p* < 0.10); ns = not significant. All models estimated by ordinary least squares (OLS) on complete cases (N = 117; no missing data). Physical activity encoded ordinally: 1 = sedentary, 2 = low, 3 = moderately active, 4 = very active. Sleep duration: 1 = < 6 h, 2 = 6–8 h, 3 = 8–10 h. Education: 0 = secondary school, 1 = university. Sex: 0 = male, 1 = female. VIF < 2.3 for all predictors (no multicollinearity). These regression models follow analytical approaches established in recent adolescent stress and somatic symptom research [9,10,11,13,14,15]. Physical activity, sleep duration, education, and meal frequency are encoded ordinally consistent with published methodology in comparable cohorts [2,10,27]. All models estimated by ordinary least squares on complete cases (N = 117).

**Table 10:** Rapid Eating Assessment for Patients (REAP): dietary behaviour frequencies (N = 117).

Dietary Behaviours	Often/Always N (%)	Sometimes N (%)	Rarely/Never N (%)	Total ≥ Sometimes N	Total %
Unhealthy Dietary Patterns					
Refined carbohydrates (white bread, pasta, white rice)	50 (42.7%)	41 (35.0%)	16 (13.7%)	91	78%
Fried food consumption (meat, fish, potatoes)	43 (36.8%)	47 (40.2%)	19 (16.2%)	90	77%
Sweets and confectionery	34 (29.1%)	41 (35.0%)	24 (20.5%)	75	64%
High-fat animal products (fatty pork, duck, beef)	33 (28.2%)	38 (32.5%)	28 (23.9%)	71	61%
Red meat > 2 times/week	34 (29.1%)	38 (32.5%)	24 (20.5%)	72	62%
Processed meat (sausages, salami, burgers)	16 (13.7%)	43 (36.8%)	42 (35.9%)	59	50%
Salty snacks (chips, crackers, popcorn with fat)	19 (16.2%)	51 (43.6%)	29 (24.8%)	70	60%
Sweetened soft drinks and juices	28 (23.9%)	40 (34.2%)	30 (25.6%)	68	58%
Between-meal snacking	29 (24.8%)	41 (35.0%)	29 (24.8%)	70	60%
Canned or smoked products > 2 times/week	11 (9.4%)	37 (31.6%)	40 (34.2%)	48	41%
Fast food ≥ 2 times/week	9 (7.7%)	15 (12.8%)	52 (44.4%)	24	21%
Breakfast skipping	30 (25.6%)	43 (36.8%)	21 (17.9%)	73	62%
Healthy Dietary Patterns					
Adequate water intake (≥1.5 L/day)	73 (62.4%)	30 (25.6%)	9 (7.7%)	103	88%
Tomatoes (fresh or cooked)	71 (60.7%)	30 (25.6%)	5 (4.3%)	101	86%
Citrus fruits (oranges, lemons, grapefruit)	51 (43.6%)	48 (41.0%)	14 (12.0%)	99	85%
Eggplant (aubergine)	53 (45.3%)	38 (32.5%)	13 (11.1%)	91	78%
Three complete meals per day	54 (46.2%)	27 (23.1%)	16 (13.7%)	81	69%
Legumes ≥ 3 times/week (beans, chickpeas, soy)	29 (24.8%)	40 (34.2%)	30 (25.6%)	69	59%
Leafy greens (spinach)	39 (33.3%)	31 (26.5%)	19 (16.2%)	70	60%
Nuts and almonds ≥ 3 times/week	27 (23.1%)	27 (23.1%)	42 (35.9%)	54	46%
Seeds (pumpkin, sunflower) ≥ 3 times/week	10 (8.5%)	24 (20.5%)	49 (41.9%)	34	29%
Dark chocolate > 60% cocoa	17 (14.5%)	42 (35.9%)	27 (23.1%)	59	50%
Mediterranean Diet Adherence Indicators					
Extra virgin olive oil	31 (26.5%)	27 (23.1%)	31 (26.5%)	58	50%
Fermented dairy (yoghurt, kefir) ≥ 3 times/week	43 (36.8%)	34 (29.1%)	22 (18.8%)	77	66%
Fresh fish ≥ 1–2 times/week	19 (16.2%)	30 (25.6%)	33 (28.2%)	49	42%
Fermented beverages (kombucha, sauerkraut juice, kimchi)	5 (4.3%)	15 (12.8%)	29 (24.8%)	20	17%
Adequate whole grain intake (≥3 portions/day) *	35 (29.9%)	32 (27.4%)	20 (17.1%)	67	57%
Beverages and Stimulants					
Coffee	52 (44.4%)	30 (25.6%)	16 (13.7%)	82	70%
Alcohol (wine, beer, spirits)	11 (9.4%)	29 (24.8%)	33 (28.2%)	40	34%
Green or black tea	23 (19.7%)	30 (25.6%)	20 (17.1%)	53	45%
Energy drinks	8 (6.8%)	22 (18.8%)	25 (21.4%)	30	26%
Eating Behaviours and Appetite					
Regular meal timing (fixed hours)	6 (5.1%)	25 (21.4%)	33 (28.2%)	31	26.5%
Appetite alteration (reduced appetite) †	44 (37.6%)	-	-	-	-
Appetite loss (screening) †	12 (10.3%)	-	-	-	-
Excessive eating (screening) †	10 (8.5%)	-	-	-	-

Often/Always and Sometimes categories combined to generate a total ≥ Sometimes prevalence. * Whole grain item phrased as frequency of eating fewer than 3 portions/day; the rarely/never response (n = 35, 29.9%) was interpreted as indicating adequate intake. Eating behaviours items (appetite loss, excessive eating) are single-item screening questions, not validated instruments. Not applicable responses excluded from percentage calculations. N = 117 for all items.

**Table 11:** Post hoc power analysis for key statistical comparisons.

Cronbach’s Alpha	N	Effect Size	Achieved Power	Assessment
Sex comparison: Total Symptom Score	105 F, 12 M	d = 0.904	0.837	Adequate
Regression Model 1: Total Symptom Score	117	R^2^ = 0.183	0.964	Adequate
Regression Model 2: Total Psychosocial Burden	117	R^2^ = 0.216	0.989	Adequate
Regression Model 3: Stigma Experienced	117	R^2^ = 0.288	1.000	Adequate
Regression Model 4: Neurological Symptom Score	117	R^2^ = 0.252	0.998	Adequate
Obese subgroup sex comparison	8 F, 1 M	-	<0.10	Severely underpowered

Note. Power calculated for two-tailed tests with *α* = 0.05 using G*Power 3.1.9.7. Regression models evaluated via F-test for overall model significance. Effect sizes: Cohen’s d for the sex-based comparison (d = 0.904, large effect); *R*^2^ (coefficient of determination) for regression models. Achieved power ≥0.80 is conventionally considered adequate. The sex comparison achieved power of 0.837 despite a large effect size, reflecting the severe sex imbalance in the sample (105 females vs. 12 males). The obese subgroup sex comparison was not conducted due to n = 1 male in the obese BMI category, rendering any inferential comparison non-informative. F = female; M = male.

## Data Availability

The data presented in this study are available upon reasonable request from the corresponding author and are not publicly available due to participant privacy considerations.

## Supplementary Materials

The following supporting information can be downloaded at: https://www.mdpi.com/article/10.3390/life16060969/s1, Supplementary Material S1: the complete survey; Supplementary Material S2: the scoring algorithm for all composite scores, including domain-level symptom scales and psychosocial burden subscales.
