# Supplementary material for: Psychosocial Burden, Multi-System Somatic Symptom Severity, and Weight-Related Stigma in Late Adolescents and Young Adults: A Cross-Sectional Survey from Romania
Source: Life (Basel). 2026 Jun 9;16(6):969. doi: 10.3390/life16060969 (PMC13301597; doi:10.3390/life16060969)
Supplement: Supplementary file 1 [file life-16-00969-s001.zip › life-4319429-supplementary2.pdf]

Supplementary Material S2

Scoring Algorithm and Variable Coding Guide for All Composite Outcome Measures

Manuscript: Psychosocial Burden, Multi-System Somatic Symptom Severity, and Weight-Related Stigma in Adolescents and Young Adults: A Cross-Sectional Survey from Romania

Authors: Raluca Maior)

Journal: Life (MDPI)

Dataset: N = 117 participants; data collected March 2025, Targu Mures, Romania

1. Overview

This document describes the complete operationalisation and scoring algorithm for all composite outcome measures used in the present study. It is organised into four sections:

- Section 2 — Somatic Symptom Domain Scales (Likert 0–5 items)
- Section 3 — Psychosocial Burden Subscales (binary items)
- Section 4 — Predictor and Covariate Coding (regression models)
- Section 5 — FDR Correction Families and Statistical Test Assignments

2. Somatic Symptom Domain Scales

2.1 Item-level response format

Each symptom item was rated by the participant on a 6-point severity scale:

- 0 = absent (no symptoms)
- 1 = very mild
- 2 = mild
- 3 = moderate
- 4 = severe
- 5 = very severe

2.2 Domain score calculation

Domain scores were calculated as the unweighted sum of all item ratings within that domain. No items were reverse-scored. Missing values were not present in the dataset (complete cases, N = 117); no imputation was applied.

Formula: Domain Score = Σ ($item_1 + item_2 + \ldots + item_n$)

2.3 Total Symptom Score

The Total Symptom Score was computed as the sum of all five domain scores:

Total Symptom Score = Digestive + Cutaneous + Respiratory + Neurological + Cardiovascular

Possible range: 0 to 185. Higher scores indicate greater somatic symptom burden.

2.4 Domain definitions, item lists, and reliability

Table S2-A below lists each domain, its constituent items, number of items, response scale, Cronbach's alpha (computed from present dataset, N = 117), and score range.

Table S2-A. Somatic Symptom Domain Scales: Item Composition, Reliability, and Score Ranges

Domain	Items included	No. Items	Scale	α	Score Range
Digestive	Bloating; gastric reflux / heartburn; nausea; vomiting; eructations; flatulence; constipation; diarrhoea; postprandial fullness; halitosis; urinary difficulties; other GI complaints (2 items)	13	0–5	0.798	0–65
Cutaneous	Eczema; facial redness / flushing; swollen or reddened eyelids; atopic dermatitis; skin pruritus; urticaria; other skin complaints	7	0–5	0.736	0–35
Respiratory	Chronic nasal congestion; frequent sneezing; rhinorrhoea; breathing difficulties / dyspnoea; prolonged cough	5	0–5	0.797	0–25
Neurological	Frequent headaches / migraines; dizziness or vertigo; brain fog (concentration difficulties, mental fatigue, word-finding difficulty); insomnia / sleep disturbances; irritability or abrupt mood changes; concentration problems; chronic fatigue	7	0–5	0.882	0–35
Cardiovascular	Palpitations; sudden blood-pressure decreases (dizziness on rising); sudden blood-pressure increases (pressure in head, tachycardia); musculoskeletal / joint pain without evident cause; excessive perspiration at rest	5	0–5	0.632	0–25
**Total Symptom Score**	**Sum of all five domain scores**	**37**	**0–5**	**0.895**	**0–185**

Note. α = Cronbach's alpha computed from complete dataset (N = 117). Interpretation: excellent ≥ 0.90; good = 0.80–0.89; acceptable = 0.70–0.79; questionable = 0.60–0.69. The cardiovascular domain (α = 0.632) reached only questionable reliability; findings for this subscale should be interpreted with additional caution. Score range = 0 to (No. items × 5).

3. Psychosocial Burden Subscales

3.1 Item-level response format

All psychosocial battery items used a binary response format:

- da (yes) = 1
- nu (no) = 0

The original questionnaire was administered in Romanian. Items presented below are provided in English translation.

3.2 Subscale score calculation

Each subscale score was calculated as the unweighted sum of binary item codes within that domain.

Formula: Subscale Score = Σ ($item_1 + item_2 + \ldots + item_n$), where each item $\in \{0, 1\}$

3.3 Total Psychosocial Burden composite score

The Total Psychosocial Burden score was defined a priori as the sum of three subscales only: Domain A (Emotional Burden), Domain B (Social Impact), and Domain C (Avoidance Behaviours). Domains D through G were analysed as separate subscales and were not included in the composite.

Total Psychosocial Burden = Domain A + Domain B + Domain C

Possible range: 0 to 12. Higher scores indicate greater psychosocial burden attributable to body weight concerns.

3.4 Subscale definitions, item lists, and reliability

Table S2-B below details all seven psychosocial domains, their constituent items, coding, Cronbach's alpha, and score ranges.

Table S2-B. Psychosocial Subscales: Item Composition, Coding, Reliability, and Score Ranges

Subscale	Items included	No. Items	Coding	α	Score Range
**Domain A: Emotional Burden**	Shame related to body weight; guilt after eating certain foods; low self-confidence due to physical appearance; social anxiety related to weight; depression or sadness due to body weight	5	da=1 nu=0	0.829	0–5
**Domain B: Social Impact**	Weight affects personal relationships; weight affects work or academic performance; weight affects participation in social activities	3	da=1 nu=0	0.822	0–3

**Domain C: Avoidance Behaviours**	Avoided beach or swimming pool due to weight; avoided gym or sports facilities; avoided photographs; avoided social events	4	da=1 nu=0	0.768	0–4
**Domain D: Stigma Experienced**	Felt discriminated against due to body weight; target of negative jokes or comments about weight	2	da=1 nu=0	0.764	0–2
**Domain E: Societal Pressure and Media**	Society pressures people to conform to appearance norms; media promotes unrealistic body image standards; social or family pressure to lose weight	3	da=1 nu=0	0.614	0–3
**Domain F: Societal Beliefs**	Overweight individuals perceived as lazier; as less intelligent; as less healthy	3	da=1 nu=0	0.803	0–3
**Domain G: Support and Resources**	Consulted psychologist about weight impact; receives family or friend support for weight management; participated in structured weight-loss programme	3	da=1 nu=0	*n/a**	0–3
**Total Psychosocial Burden (A+B+C)**	**Sum of Domains A, B, and C only**	**12**	**da=1 nu=0**	**0.889**	**0–12**

Note. α = Cronbach's alpha computed from complete dataset (N = 117). n/a = Domain G (Support and Resources) was not included in any composite score and was analysed only at the item level (frequencies); alpha not reported. Societal Pressure and Media (Domain E) reached only questionable reliability (α = 0.614); findings for this subscale should be interpreted with additional caution. Total Psychosocial Burden includes Domains A+B+C only (12 items, α = 0.889).*

4. Predictor and Covariate Coding

Table S2-C details the operationalisation and encoding of all predictor and covariate variables entered simultaneously into the four multivariable ordinary least squares (OLS) regression models reported in Table 6 of the main manuscript.

Table S2-C. Predictor and Covariate Variables: Operationalisation and Encoding

Variable	Operationalisation	Scale / Values	Encoding in analyses	Type
BMI	Self-reported weight (kg) ÷ height (m)²	Continuous	Continuous in correlation and regression; categorical (underweight / normal / overweight / obese) in Kruskal–Wallis	Continuous / Ordinal
Perceived Stress	Single-item Likert rating of current stress level	1–5	Continuous (ordinal treated as interval) in correlation and regression	Ordinal
Sex	Self-reported biological sex	Female / Male	Binary dummy: female = 1, male = 0	Binary
Age	Self-reported age in completed years	16–20 years	Continuous in regression	Continuous
Physical Activity Level	Self-reported activity level (4-point scale)	1=Sedentary 2=Low	Ordinal (1–4) in regression	Ordinal

		3=Moderate 4=Very active		
Sleep Duration	Self-reported usual nightly sleep duration	1=<6 h 2=6–8 h 3=8–10 h	Ordinal (1–3) in regression	Ordinal
Education Level	Highest education level completed or in progress	Secondary / University	Binary dummy: university = 1, secondary = 0	Binary
Daily Meal Frequency	Self-reported number of meals per day	1=1–2 meals 2=3 meals 3=4–5 meals	Ordinal (1–3) in regression	Ordinal

Note. All eight predictors were entered simultaneously in each regression model (forced entry). No stepwise or backward elimination procedures were used. Variance inflation factors (VIF) were below 2.3 for all predictors in all models, indicating the absence of problematic multicollinearity. BMI categories for Kruskal–Wallis analyses followed WHO adult criteria: underweight < 18.5; normal weight 18.5–24.9; overweight 25.0–29.9; obese ≥ 30.0 kg/m².

5. Multiple Testing Correction: FDR Families

False Discovery Rate (FDR) correction was applied using the Benjamini–Hochberg procedure separately within four pre-defined test families. Table S2-D below specifies each family, the tests included, and the number of comparisons corrected.

Table S2-D. Definition of FDR Correction Families

Family	Tests included	Outcomes tested	No. tests	Test type
1	BMI (continuous) vs. all outcome variables	5 symptom domains + TSS + 4 psychosocial subscales + Total Psych. Burden	11	Spearman rS
2	Perceived Stress vs. all outcome variables	5 symptom domains + TSS + 4 psychosocial subscales + Total Psych. Burden	11	Spearman rS
3	BMI category differences in all outcomes	5 symptom domains + TSS + 4 psychosocial subscales + Total Psych. Burden	11	Kruskal–Wallis H
4	BMI category vs. individual binary perception items (chi-square)	17 binary psychosocial perception items	17	Chi-square χ^2
**Total**			**50**	

Note. FDR correction applied using the Benjamini–Hochberg procedure within each family independently. Both raw p-values (praw) and FDR-adjusted p-values (pFDR) are reported in all results tables of the main manuscript. TSS = Total Symptom Score. Reference: Benjamini, Y.; Hochberg, Y. Controlling the false discovery rate: A practical and powerful approach to multiple testing. J. R. Stat. Soc. B 1995, 57, 289–300.

6. Software

All scoring, coding, and statistical analyses were implemented in Python 3.12 using the following libraries: pandas 2.2 (data management and score computation); SciPy 1.13 (Spearman correlations, Mann–Whitney U, Kruskal–Wallis, chi-square, Shapiro–Wilk normality tests); statsmodels 0.14 (ordinary least squares regression, VIF computation); pingouin 0.5 (Cronbach's alpha, epsilon-squared effect sizes). Statistical significance threshold: α = 0.05 (two-tailed) for all tests.
